# Supplementary material for: Transcriptome Analysis Reveals Vimentin-Induced Disruption of Cell–Cell Associations Augments Breast Cancer Cell Migration
Source: Cells. 2022 Dec 13;11(24):4035. doi: 10.3390/cells11244035 (PMC9776984; doi:10.3390/cells11244035)
Supplement: Supplementary file 1 [file cells-11-04035-s001.zip › DEGs and enrichment analysis.zip!/DEGs and enrichment analysis/WT_VIMvsVector GO enrichment/GSEA/WT_VIM/INTERMEDIATE_FILAMENT_ORGANIZATION/INTERMEDIATE_FILAMENT_ORGANIZATION(GO_0045109).html]

Details for gene set INTERMEDIATE\_FILAMENT\_ORGANIZATION(GO:0045109)[GSEA]


|  || Dataset | fpkm.sample |
| Phenotype | sample.cls |
| Upregulated in class | WT\_VIM |
| GeneSet | INTERMEDIATE\_FILAMENT\_ORGANIZATION(GO:0045109) |
| Enrichment Score (ES) | 0.9509824 |
| Normalized Enrichment Score (NES) | 1.45948 |
| Nominal p-value | 0.0 |
| FDR q-value | 0.16299972 |
| FWER p-Value | 0.163 |
Table: GSEA Results Summary

  

Fig 1: Enrichment plot: INTERMEDIATE\_FILAMENT\_ORGANIZATION(GO:0045109)
  
Profile of the Running ES Score & Positions of GeneSet Members on the Rank Ordered List

  

| PROBE | DESCRIPTION   (from dataset) | GENE SYMBOL | GENE\_TITLE | RANK IN GENE LIST | RANK METRIC SCORE | RUNNING ES | CORE ENRICHMENT || 1 | ENSG00000175084 | DES |  |  | 2208 | Infinity | -0.0373 | Yes |
| 2 | ENSG00000026025 | VIM |  |  | 2388 | 1709.539 | 0.9510 | Yes |
| 3 | ENSG00000081277 | PKP1 |  |  | 4663 | 2.579 | 0.9140 | No |
| 4 | ENSG00000100285 | NEFH |  |  | 6771 | 1.860 | 0.8794 | No |
| 5 | ENSG00000171403 | KRT9 |  |  | 7795 | 1.660 | 0.8631 | No |
| 6 | ENSG00000164690 | SHH |  |  | 8548 | 1.541 | 0.8513 | No |
| 7 | ENSG00000186847 | KRT14 |  |  | 11204 | 1.247 | 0.8071 | No |
| 8 | ENSG00000057294 | PKP2 |  |  | 12177 | 1.167 | 0.7913 | No |
| 9 | ENSG00000173744 | AGFG1 |  |  | 12722 | 1.123 | 0.7828 | No |
| 10 | ENSG00000131095 | GFAP |  |  | 14245 | 1.007 | 0.7576 | No |
| 11 | ENSG00000096696 | DSP |  |  | 19375 | 0.721 | 0.6713 | No |
| 12 | ENSG00000171100 | MTM1 |  |  | 20482 | 0.662 | 0.6529 | No |
| 13 | ENSG00000105993 | DNAJB6 |  |  | 20888 | 0.641 | 0.6465 | No |
| 14 | ENSG00000128422 | KRT17 |  |  | 25113 | 0.336 | 0.5752 | No |
| 15 | ENSG00000114796 | KLHL24 |  |  | 25534 | 0.305 | 0.5683 | No |
| 16 | ENSG00000172867 | KRT2 |  |  | 29036 | 0.000 | 0.5090 | No |
| 17 | ENSG00000139648 | KRT71 |  |  | 33878 | NaN | 0.4272 | No |
| 18 | ENSG00000104722 | NEFM |  |  | 50333 | NaN | 0.1488 | No |
| 19 | ENSG00000204897 | KRT25 |  |  | 55079 | NaN | 0.0686 | No |
| 20 | ENSG00000277586 | NEFL |  |  | 56226 | NaN | 0.0492 | No |
| 21 | ENSG00000171431 | KRT20 |  |  | 59046 | NaN | 0.0015 | No |
Table: GSEA details
[plain text format]

  

Fig 2: INTERMEDIATE\_FILAMENT\_ORGANIZATION(GO:0045109)
  
Blue-Pink O' Gram in the Space of the Analyzed GeneSet

  

Fig 3: INTERMEDIATE\_FILAMENT\_ORGANIZATION(GO:0045109): Random ES distribution
  
Gene set null distribution of ES for
 **INTERMEDIATE\_FILAMENT\_ORGANIZATION(GO:0045109)**
